# The Role of Nuclear Fragmentation in Particle Therapy and Space Radiation Protection

**DOI:** 10.3389/fonc.2016.00065

**Published:** 2016-03-29

**Authors:** Cary Zeitlin, Chiara La Tessa

**Affiliations:** ^1^Lockheed Martin Information Services & Global Solutions, Houston, TX, USA; ^2^Collider-Accelerator Department, Brookhaven National Laboratory, Upton, NY, USA

**Keywords:** galactic cosmic rays, nuclear fragmentation models, nuclear interactions, Bragg curve, space radiation, space radiation shielding, heavy-ion therapy

## Abstract

The transport of the so-called HZE particles (those having high charge, *Z*, and energy, E) through matter is crucially important both in space radiation protection and in the clinical setting where heavy ions are used for cancer treatment. HZE particles are usually considered those having *Z* > 1, though sometimes *Z* > 2 is meant. Transport physics is governed by two types of interactions, electromagnetic (ionization energy loss) and nuclear. Models of transport, such as those used in treatment planning and space mission planning must account for both effects in detail. The theory of electromagnetic interactions is well developed, but nucleus–nucleus collisions are so complex that no fundamental physical theory currently describes them. Instead, interaction models are generally anchored to experimental data, which in some areas are far from complete. The lack of fundamental physics knowledge introduces uncertainties in the calculations of exposures and their associated risks. These uncertainties are greatly compounded by the much larger uncertainties in biological response to HZE particles. In this article, we discuss the role of nucleus–nucleus interactions in heavy charged particle therapy and in deep space, where astronauts will receive a chronic low dose from galactic cosmic rays (GCRs) and potentially higher short-term doses from sporadic, unpredictable solar energetic particles (SEPs). GCRs include HZE particles; SEPs typically do not and we, therefore, exclude them from consideration in this article. Nucleus–nucleus collisions can result in the breakup of heavy ions into lighter ions. In space, this is generally beneficial because dose and dose equivalent are, on the whole, reduced in the process. The GCRs can be considered a radiation field with a significant high-LET component; when they pass through matter, the high-LET component is attenuated, at the cost of a slight increase in the low-LET component. Not only are the standard measures of risk reduced by fragmentation, but it can be argued that fragmentation also reduces the uncertainties in risk calculations by shifting the LET distribution toward one that is more concentrated at low LET, where biological effects are better understood. We review previous work in this area, including measurements made by the Radiation Assessment Detector during its journey to Mars and while on the surface of Mars aboard the Curiosity rover. Transport of HZE is also critically important in heavy-ion therapy, as it is necessary to know the details of the radiation field at the treatment site. This field is substantially modified compared to the incident pure (or nearly pure) ion beam by the same mechanisms of energy loss and nuclear fragmentation that pertain to the transport of space radiation.

## Introduction

The situation for cancer treatment with beams of heavy charged particles is quite different from that in space, but there is important overlap between the transport physics in the two settings. In the clinic, dose localization is of paramount importance, but nuclear fragmentation degrades localization. By contrast, the same process is beneficial in space, because high-LET particles are broken up into particles with lower LET and (in most cases) reduced biological effectiveness.

Fragmentation significantly complicates treatment planning, because the lower-LET particles produced in these reactions have greater ranges than the primary beam ions and, therefore, deposit energy beyond the distal edge of the Bragg peak. Low-LET particles may also be produced at significant angles with respect to the incoming beam, resulting in a lateral leakage of dose into healthy tissues. Target fragments, which consist of short-ranged, high-LET charged particles and neutrons, are emitted more or less isotropically from the struck nucleus, and may cause very large energy deposits anywhere along the path of the incident ion. Such reactions are particularly undesirable when they occur in the entrance region, since they tend to undermine one of the primary benefits of heavy ions for therapy, the large peak-to-plateau dose ratio. Target fragments are also produced by proton beams and complicate treatment planning in that modality (1).

These effects, particularly the irradiation of healthy tissues beyond the distal edge of the Bragg peak, effectively limit the maximum ion charge that can be used in treatment. For any given beam ion species, and any given depth of treatment volume, it is possible to find a beam energy that will yield a Bragg peak in the desired location. This might seem to suggest that the highest possible *Z* should be used, in order to maximize the peak-to-plateau ratio of biological dose. However, the distal edge problem worsens significantly with increasing beam charge. In the early days of heavy-ion therapy at the Lawrence Berkeley Laboratory’s Bevalac, ions as heavy as neon (*Z* = 10) were used. Current practice in Japan and Europe has largely been focused on carbon ions (*Z* = 6) as representing a more optimal trade. Helium ions (*Z* = 2, mass number 4) are also of considerable interest, and our analysis of recently obtained cross-sectional data suggests that they may provide a localization advantage. This arises from the fact that the nucleons in helium nuclei are especially tightly bound, making them relatively less likely to fragment. When they do undergo fragmentation, the most copiously produced isotope is ^1^H, which for a given kinetic energy per nucleon has almost exactly the same range as ^4^He. This fact significantly reduces the distal edge problem, though deuterons (^2^H) and neutrons produced in fragmentation reactions do deposit some energy in the distal edge.

In space, exposure to heavy ions increases cancer risk; in medicine, the same ions may be used to treat cancer. In the following, we will compare and contrast the effects of nuclear fragmentation in these two environments. An extensive literature on nuclear reactions relevant to spaceflight exists, and was recently reviewed by Norbury et al. (2) Since the beginning of human spaceflight, it has been recognized that energetic charged particles pose a health risk to explorers. When mission durations were short, on the order of hours or days and confined to low-Earth orbit (LEO), the main concerns were exposure to large solar-particle events (SPEs) and trapped radiation. SPEs, which typically produce protons with kinetic energies below 100 MeV, are a concern even on short missions for two reasons: first, because they can produce high dose rates, particularly in situations where shielding is minimal, and, second, because they are unpredictable and sometimes have sudden onsets (3). In LEO, there is geomagnetic shielding of galactic cosmic rays (GCRs) and also partial blocking of particle fluxes by the Earth (roughly a 35–40% effect, depending on altitude). Exposure to GCRs in LEO gives a small but steady dose rate, on the order of 100–300 μGy/day depending on the phase of the solar cycle; in such orbits, there is a roughly equal contribution from trapped radiation, so that in the absence of SPEs, total doses are well under 1 mGy/day. Considering that, in the ICRP 60 formulation (4), the average radiation quality factor in space is in the range from 6 to 7 (less behind shielding), this leads to exposures of <5 mSv/day in terms of dose equivalent. Although this is a far higher rate than encountered on Earth (about 4 mSv/year average in the United States), such exposures are not of concern for mission durations of a few days or weeks – they are far below threshold for acute effects, and small enough that they would not be expected to significantly increase lifetime fatal cancer risk.

The exposure scenario for long-duration missions into deep space is considerably different from those of short-duration missions to LEO or even to the Moon as in the Apollo era. Deep-space missions of the future are likely to be longer in duration than most if not all missions to date, with modestly shielded vehicles, and by definition will be outside the (partial) protection of LEO. In long-duration mission scenarios, the dominant radiation health risks are almost certain to be those from GCRs, including a significant component of heavy ions (5). Because GCRs tend to be highly energetic, most of them pass through the moderate shielding (probably on the order of 20 g cm^−2^) that is to be expected on a vehicle built for crewed travel into deep space. Exposure to energetic heavy ions is unavoidable; however, shielding does attenuate the heavy ion flux due to nuclear interactions that cause the incident ions to fragment into lighter ions. Choosing shield materials to maximize nuclear fragmentation is at present the most viable strategy for reducing this exposure, though there is certainly a shielding depth at which the law of diminishing returns begins to pertain. Other approaches, including magnetic and electrostatic shielding, are not yet practical, nor is it feasible (from the cost perspective) to launch shields consisting of hundreds of gram per square centimeter of mass, or even many tens of gram per square centimeter.

In the following, we will review transport physics as it pertains to energetic charged particles encountered in space and used in radiation therapy, with particular emphasis on the unique roles played by nuclear fragmentation in these two very different settings. A brief overview of fragmentation models is also given. We will present both data and model calculations to support the conclusions outlined here, both for space radiation and for beams of interest in the clinic. It should be added that proton–nucleus collisions are also extremely important in both settings, but are not covered in detail here.

## Transport of Energetic Charged Particles

The transport of energetic particles through spacecraft walls, equipment racks, and human tissues determines the physical dose received by astronauts in space, and the same physical mechanisms affect the beams of charged particles that are used to irradiate tumors. When charged particles traverse matter, electromagnetic interactions cause ionization energy loss, which continuously slows the incident particles, increasing their LET. These interactions are between the electromagnetic field of the projectile and the electrons surrounding the atoms in the material being traversed. The projectiles considered here are bare nuclei, fully stripped of all electrons. These interactions produce a region of relatively dense ionization along the trajectory of the projectile and can also result in the production of long-ranged, high-energy “knock-on” electrons, also known as δ-rays, which can deposit dose at a considerable distance from the main track. Detailed models of track structure (6–8) describe these complexities, which include energy deposition at significant distances from the primary track in the plane transverse to the direction of the incident particle. Electromagnetic interactions also cause Coulomb multiple scattering, but for the particles and energies of interest here, we are for the most part not concerned with this process as it produces mostly very small angle deflections. The interested reader is referred to the literature (9).

The nuclear interactions of interest span a broad range of possibilities, from highly peripheral interactions in which only a small number of nucleons are removed from the projectile to central collisions in which the incoming projectile is fragmented into a high-multiplicity spray of light ions. Also of interest in nuclear interactions is the production of target fragments, including neutrons that are capable of penetrating large depths of matter before interacting.

### Ionization Energy Loss

**Figure 1:**
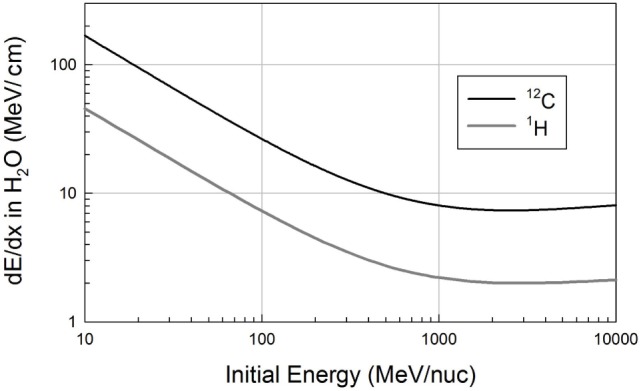
**Ionization energy loss curves for protons (^1^H) and carbon ions in water, calculated from the Bethe equation**.

Ionization energy loss is a purely electromagnetic phenomenon in which a charged projectile interacts with the electrons in the atoms of the target medium. The energy lost by the projectile per unit path length is accurately described by the Bethe equation (9):

$$\frac{\text{dE}}{\text{dx}}=-k\rho {\left(\frac{Z}{A}\right)}_{\text{mat}}{\left(\frac{Z}{\beta }\right)}_{\text{proj}}^{2}\left[\log\left(\frac{2m_{\text{e}}\beta^{2}}{I\left(1-\beta^{2}\right)}\right)-\beta^{2}\right]$$

where k is a constant, *Z* refers to atomic number, *A* to mass number, β is the velocity of the moving particle relative to the speed of light, *I* is the ionization potential of the medium, and ρ its density. The subscript “mat” refers to the material being traversed (often referred to as the target, here as the shield), while “proj” refers to the projectile. The density effect, neglected in the equation above, is applicable at high energy, and additional corrections are needed at very low energies, where the curve turns over as particles approach their stopping points. The term in brackets is slowly varying with projectile energy, so that for moderate energies (β not too close to 1), dE/dx goes as (*Z*^2^/β^2^) of the projectile, and as (*Z*/*A*) of the target material. Integrating the dE/dx vs. energy curve yields the range–energy relation for any combination of projectile and target. To a very good approximation, the proton range for a given energy and material can be scaled to obtain the range of an ion with the same velocity (or energy per nucleon) having charge *Z* and mass number *A* according to (*A*/*Z^*2*^*). Calculations of dE/dx have been shown to be highly accurate (typically to better than 1%) over a wide range of projectiles and targets; the main uncertainties are the ionization potentials. This part of the transport problem is well understood and can be modeled with high confidence. The dependence on *Z*^2^ and energy can be seen in Figure 1, which shows straightforward dE/dx vs. energy calculations for ^12^C ions and protons in water for kinetic energies of 10 MeV/nuc and above (the region of interest for space applications). The curves are approximately flat at high energies, but rise significantly at the lower energies that are especially relevant for hadron therapy.

### Nuclear Interactions

The electromagnetic interactions described in the preceding section are well understood from both theoretical and experimental perspectives. Nuclear interactions are also a crucial aspect of transport, but are not nearly as well understood from the theoretical perspective. Interactions between a projectile and atoms of the target are ultimately described by quantum electrodynamics (QED), the most accurate physical theory yet devised; by contrast, nuclear interactions are many-body problems that defy present-day calculational methods at the most fundamental level. That is, the particles that participate in nuclear interactions are themselves composites (nuclei contain nucleons, and nucleons contain quarks and gluons), and the fundamental theory that describes these interactions is quantum chromodynamics (QCD), which is only tractable in the limit of interactions with large momentum transfers. QCD has not yet been successfully applied to nucleus–nucleus collisions at the energies of interest here. The lack of a fundamental theory has led to the development of many semi-empirical models to describe nucleus–nucleus interactions, and considerable effort continues to be put into development of these models and benchmarking them (10) against the limited set of pertinent data that are available (2).

Outgoing particles from a heavy-ion fragmentation reaction are typically described as either “projectile” fragments or “target” fragments. Projectile fragments approximately preserve the direction and velocity of the incident particle. Target fragments are produced when the nuclei in the medium being traversed participate in an interaction, and they or their remnants are left in an excited state. These states decay via emission of nucleons, including neutrons. Target fragments are emitted more or less isotropically in the laboratory frame, and have relatively low energies, on the order of tens of MeV or less. A nucleus–nucleus collision can, if it is central (i.e., head-on), produce a large multiplicity of projectile fragments, each of which has lower LET than did the incident ion, owing to the fact that dE/dx goes roughly as (*Z/*β)^2^, and here β is roughly constant while *Z* decreases. The sum of the LETs of the projectile fragments is always less than the LET of the primary ion. Charged target fragments can have very high LETs, but have very short ranges. The typical target-fragment energy range is also the range in which the radiation weighting factor for neutrons is highest. Neutrons can also be produced as projectile fragments, stripped from the projectile.

### Nuclear Cross Sections and Bragg Curves

Cross sections for nucleus–nucleus interactions that produce a charge change in the projectile are accurately described by a simple energy-independent model of overlapping spheres, as first postulated by Bradt and Peters (11). Wilson and Townsend (12) presented a slightly modified version for use in NASA space radiation transport codes:

$$\sigma_{\text{cc}}=\pi r_{0}^{2}{\left(A_{\text{proj}}^{1/3}+A_{\text{targ}}^{1/3}-0.2-1/A_{\text{proj}}-1/A_{\text{targ}}\right)}^{2}$$

where σ*_*cc*_* is the charge-changing cross section, the *A* values refer to the mass numbers of the projectile and target, and *r*_0_ is the nucleon radius, which is known from other data to be roughly 1.2–1.5 fm. Several other variations on the basic Bradt–Peters model exist (13, 14), but all yield similar results. As will be discussed below, the Wilson–Townsend formula reproduces measured charge-changing cross sections over a wide range of projectile and target masses, for energies from a few hundred MeV/nuc to at least 1 GeV/nuc. We can use data to constrain *r*_0_ and also to investigate the “nuclear transparency” term in the above formula, which is represented by a constant with value −0.2. This term corresponds to the probability that the spheres overlap without causing the projectile to lose charge; representing this probability by a simple constant may be an oversimplification.

**Figure 2:**
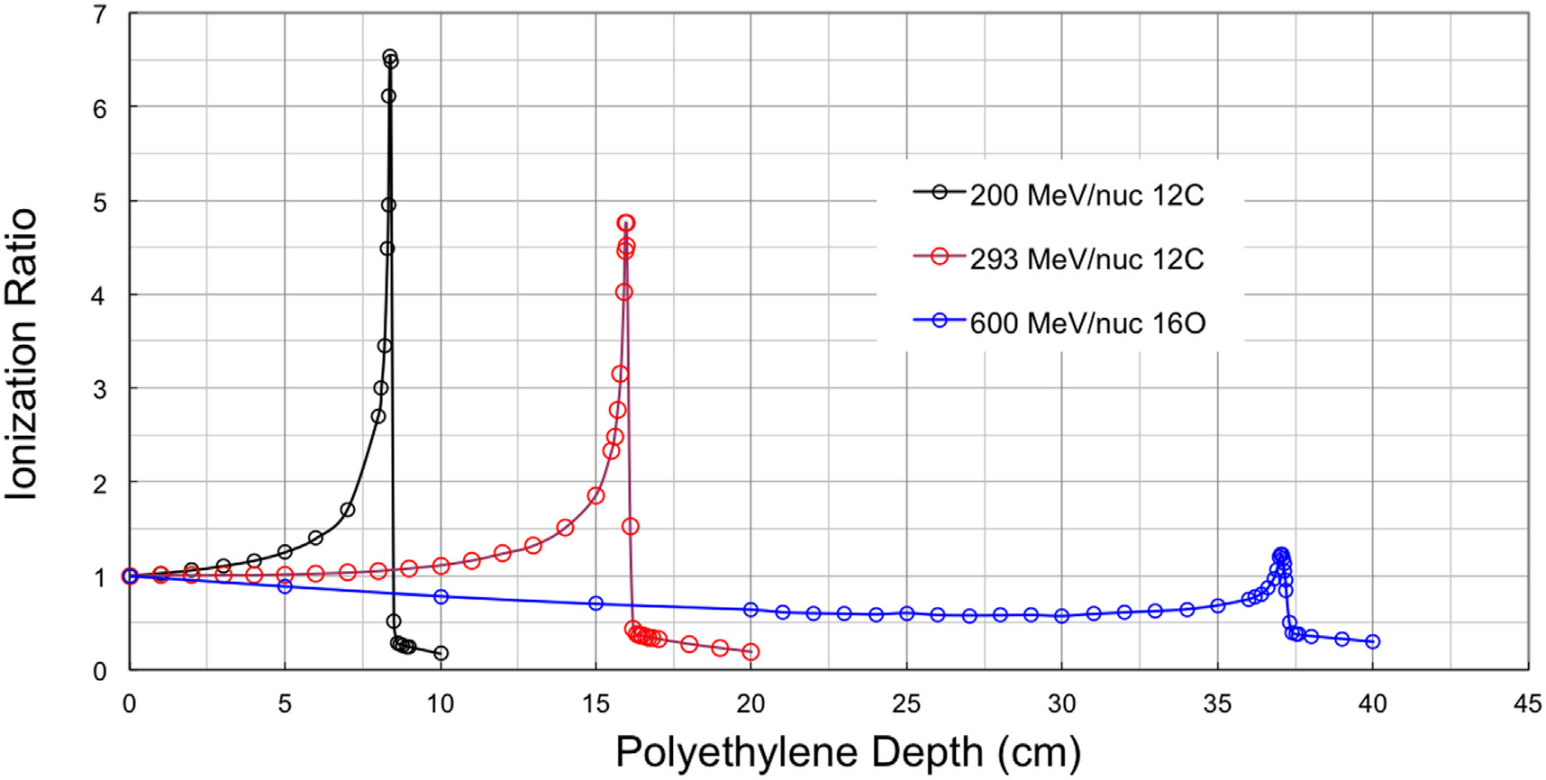
**Bragg curve data from NSRL for three beams**.

Measured Bragg curves obtained with monoenergetic ion beams illustrate the competing effects of fragmentation and ionization energy loss. Figure 2 shows depth-dose curves obtained for three different beams at the NASA Space Radiation Laboratory (NSRL) (15). The NSRL is a dedicated NASA facility at the Brookhaven National Laboratory. The Bragg curve data are publicly available on the NSRL web site. High-density polyethylene (CH_2_, with density ρ = 0.97 g cm^−3^) was used as a moderator and parallel-plate ionization chambers were used to record the relative ionization before and after the moderator; the ratio of the two (after to before) is plotted on the *y*-axis. The shortest-range beam of the three considered here is the 200 MeV/nuc ^12^C, which penetrates to a depth of about 8.4 cm of the target material before stopping. The Bragg curve increases relatively quickly with increasing target depth due to the low energy of the primary beam ions; this case is dominated by energy loss. At 293 MeV/nuc, ions of the same species travel nearly twice as far compared to the 200 MeV/nuc ions, and the Bragg curve shows a slower rise and a smaller peak. This is because the initial dE/dx is lower at the higher energy, and also because fragmentation of the primary beam ions begins to exert a significant influence. Using published data (16), we estimate that ^12^C ions in polyethylene of this density have an interaction mean free path of about 23 cm. The fraction of surviving primaries at depth *x* is given by *e*^−x/λ^ where λ is the interaction mean free path (mfp), so that over the first 12 cm of the target, some 40% of them undergo a charge-changing interaction, and at the Bragg peak (16 cm), roughly 50% of them have fragmented. About 20% of these interactions produce boron fragments (*Z* = 5), which have slightly longer ranges than the carbon primaries, and somewhat lower LET. These contribute to the distal edge just past the Bragg peak, while lighter fragmentation products – dominantly ^4^He ions – give a non-negligible dose for several centimeter beyond the Bragg peak. The distal edge dose is also apparent for 200 MeV/nuc ^12^C, but is not nearly as prominent because a smaller fraction of primary ions undergo fragmentation before reaching the Bragg peak.

The Bragg curve for 600 MeV/nuc ^16^O is also shown in Figure 2, primarily to show a contrasting case in which fragmentation dominates over ionization energy loss. For this beam, dE/dx is initially relatively low and (compared to the lower-energy beams) in a relatively flat portion of the dE/dx curve. The Bragg peak, therefore, occurs deep in the target, at about 37 cm. Again using published data, the mfp for ^16^O to undergo a charge-changing interaction in CH_2_ of this density is found to be about 17 cm, so that the survival fraction of primaries at the ionization peak is only 11%. The remaining mix of particles has comparatively low LET, so that the peak ionization barely surpasses the initial value at the entrance. Over most of the target depth, the 600 MeV/nuc ^16^O beam produces ionization ratios <1, that is, less than that of the initial, unfragmented beam. We will return to the subject of these Bragg curves in the discussion of fragmentation models below.

#### Projectile Fragments

Projectile fragments retain, to a large degree, the velocity and direction of the projectile, which makes intuitive sense considering that they are essentially intact pieces of the incident nucleus. However, velocities and directions are not exactly preserved, and the deviations are especially important in the clinical setting where these changes may contribute to dose outside the treatment volume. The changes of momentum of projectile fragments compared to the primary are mostly well-described by the statistical theory of Goldhaber (17). Projectile fragments in general have shifts in both transverse and longitudinal momentum; the change in each Cartesian coordinate is normally distributed, i.e., the probability distribution goes as exp(−*p*^2^/2σ^2^) where $\sigma^{2}=\sigma_{0}^{2}A_{\text{frag}}\left(A_{\text{proj}}-A_{\text{frag}}\right)/\left(A_{\text{proj}}-1\right)$ and σ_0_ is on the order of 90 MeV/c. Goldhaber’s work elegantly derives these relationships in two independent ways: from considerations of the Fermi motion of the nucleons inside the projectile nucleus prior to the collision, and also from thermal equilibrium, with σ_0_ directly related to the equilibrium temperature. The theory was developed to explain the momentum distributions measured in nuclear emulsion by Heckman et al. (18), and has been validated with more recent data (19–21) using an indirect method. In the latter work, σ_0_ was tuned to match individual data sets, and the model was then used to make essential corrections for angular acceptance, enabling extraction of light-fragment production cross sections from measurements made at 0° with small-acceptance detectors. These cross sections probe central (i.e., head-on) collisions, whereas the more common peripheral interactions tend to produce fragments with a small charge change from the primary.

As previously mentioned, in the clinical setting, fragmentation reactions lead to undesirable results, because fragments do not necessarily deposit their energy in the treatment volume. A qualitative assessment can be made using the formulas above. Given a ^12^C projectile and (as is commonly produced) a ^4^He fragment, the width of the momentum distribution in one transverse dimension is about 38 MeV/c, and in the two transverse dimensions is 54 MeV/c. If an interaction occurs near the entrance, when the primary has a kinetic energy of, for example, 250 MeV/nuc, then the longitudinal momentum of the fragment is roughly 3 GeV/c, so that the distribution of the polar angle has a width of about 1°. Since the distribution is normal, 95% will be contained within a cone of 2° width, 99.7% within 3°, etc. If the distance between the interaction point and the nominal stopping point of the primary is 50 mm, then deflections of 2° or greater produce transverse offsets of 1.7 mm or greater. For particles starting in the center of the beam, fragments undergoing such small deflections may remain within the treatment volume, but some particles starting near the edge of the beam will produce fragments that deposit energy outside the desired volume. This behavior is readily modeled, as is Coulomb scattering, as described below. Fragmentation can be thought of as creating a halo of projectile fragments that will tend to smear out what would otherwise be a sharp lateral edge defined by the beam.

The fragmentation of ^12^C into helium produces dose in the far distal edge of the Bragg curve. A reasonably accurate estimate of this effect can be deduced from elementary considerations (22). Closer to the Bragg peak, other fragment species contribute, but hydrogen and helium are the only ions that can penetrate far past the Bragg peak.

#### Target Fragments

**Figure 3:**
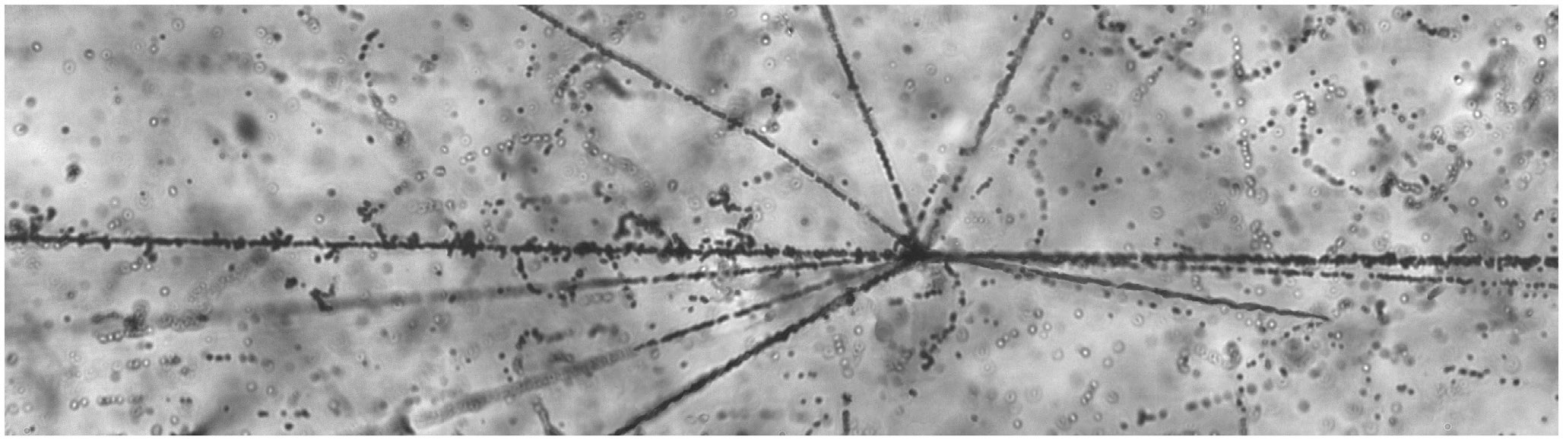
**A nuclear interaction “star” seen in photographic emulsion**. The ^28^Si beam ion is incident from the left. The backward-going tracks are target fragments.^1^ ^1^The photomicrograph used in Figure 3 was made available by P. Zarubin et al. It, and many others, are available online at http://becquerel.jinr.ru/movies/movies.html

Empirical understanding of the composition of GCRs and of nuclear fragmentation owes much to work done with nuclear emulsions flown to high altitudes in the late 1940s and 1950s (23). Using a visual detection medium allows for the observation of short-ranged, high-LET target fragments emerging from interaction vertices, sometimes referred to as “stars.” An example of an interaction vertex is shown in Figure 3, in which a 130 MeV/nuc ^28^Si beam ion (incident from the left) interacts with a heavy nucleus in the emulsion. These fragments are difficult to detect by other means, but they are important in that they produce very large, localized energy depositions in the vicinity of the interaction point. Both charged fragments and neutral particles (which, unlike the charged fragments, may penetrate long distances) can emerge from the remnants of the struck nucleus, which may in the immediate aftermath of the collision be in an excited state, from which it decays to a ground state via particle emission. Target fragments are emitted isotropically in the rest frame of the target, which to a good approximation is also the laboratory frame. In addition to the local energy depositions from charged fragments, the production of neutrons may be dosimetrically important, as their subsequent interactions can produce additional high-LET secondaries. In the context of radiation therapy, these may occur outside the tumor volume; in the context of space radiation, target-fragment neutrons produced in spacecraft walls can reach inhabited areas and contribute to crew exposures.

### High Level Overview of Models

Monte Carlo codes, such as GEANT4 (24), MCNPX (25), FLUKA (26), and PHITS (27), have been developed by the high-energy and nuclear physics communities to model the transport of ions through matter. In the space radiation protection community, the same codes are used, along with an analytic transport model known as HZETRN that was developed within NASA by Wilson et al. (28) and subsequently extended (29). In the Monte Carlo codes, particles are followed in small steps through the medium, and the relevant physical processes (ionization energy loss, Coulomb scattering, and, for ions, nuclear interactions) are simulated in each step. The analytic approach, based on numerical solution of the Boltzmann equation, yields faster computation times in some cases, but many approximations are required, and these may compromise the accuracy of the results. For some purposes, such as spacecraft design, high accuracy is not needed in the initial stages and analytic calculations may suffice, but this approach is not applicable to treatment planning. Monte Carlo codes generally require considerable effort to define the geometry of the target (e.g., a large detector, a spacecraft, or a human in a therapy beam), and may require relatively long run times depending on the complexity of the geometry.

For either space radiation transport or treatment planning in a heavy ion beam, a nuclear physics model is needed, regardless of whether the analytic or Monte Carlo approach is used. The diversity of models likely accounts for the differences observed between codes (10, 30). In some of the Monte Carlo codes, there are also a variety of options available, i.e., the user selects a particular nuclear interaction model or models. Unlike HZETRN, the Monte Carlo codes do transport calculations in three dimensions, and are inherently capable of capturing important details of nuclear reactions that are lost in one-dimensional transport.

In the following sections, we use the PHITS Monte Carlo code to illustrate the important effects of shielding in modifying GCR fields (see Nuclear Interactions and Shielding in Space) and to compare calculated Bragg curves to the NSRL Bragg curve data (see Nuclear Interactions of Carbon Beams). These comparisons allow us to demonstrate some of the important capabilities of Monte Carlo codes vis-a-vis heavy ion transport, at least in relatively simple beamline geometry. This is not intended to constitute an endorsement of PHITS, but rather reflects our previous use of the code in similar geometries. Though the goal of reproducing Bragg curves may seem straightforward, it is in fact challenging to model the experimental results with high precision. And while not all aspects are perfectly reproduced, the relatively good agreement with the data gives us confidence that the model represents the mixtures of primaries and fragments that are present before, in, and beyond the Bragg peaks, providing insights that are not available from the data alone. It is highly likely that similar results would be obtained using the other Monte Carlo codes mentioned above.

## Nuclear Interactions and Shielding in Space

In space, protons and high-charged nuclei undergo nuclear interactions as they traverse the hull of a spacecraft and the equipment inside. These are the same types of interactions that occur in the treatment setting as particle beams traverse healthy tissues on the way to the target volume. Nuclear interactions may produce a large number of secondary particles, particularly when incident energies are large. Shielding generally reduces the hazard from heavy ions due to the effects of nuclear fragmentation. Although heavy ions (those with charge Z > 2) represent only about 1% of the GCR flux, their contribution to dose in unshielded space can be 30 to 40% of the total. This disproportionate contribution can be understood by recalling that energy loss (which is directly related to dose imparted) is proportional to *Z^*2*^*, that is, to the square of the projectile’s charge. The dose-weighted average charge of GCR heavy ions is about 10, so that the dose per particle is roughly 100 times greater than that of a proton having the same kinetic energy per nucleon. The contributions of GCR heavy ions to dose equivalent are even larger than their contributions to dose, owing to the large factors by which their fluxes are weighted. In free space, iron ions (*Z* = 26 and average LET about 155 keV/μm in water) make the largest contribution of any single ion species, despite being less abundant than protons by nearly four orders of magnitude.

Given that spacecraft to date have been constructed with aluminum hulls, and given our knowledge of the fragmentation cross sections of many ion species at typical GCR energies, we can estimate how much the fluxes of various primary ions are attenuated by fragmentation before passing through a hull. Table 1 shows the results for several important species using cross sections calculated with the Townsend and Wilson energy-independent formula given above. Note that for the lighter ions, we expect there to be some replenishment by fragmentation of heavier ions (e.g., Fe + Al → Si + X, etc.); this is discussed further in Section “Measurements and Calculations for Space” below. There is also attenuation due to ionization energy loss, particularly at the larger depths, as will also be shown below. It is notable that 20 g cm^−2^ of aluminum is sufficient to break up the majority of incident iron ions and roughly half of magnesium and silicon ions.

**Table 1:** **Calculated attenuation of high-energy ions by fragmentation in aluminum using geometric cross sections**.

Ion	5 g cm^**−**2^ Al	10 g cm^**−**2^ Al	20 g cm^**−**2^ Al	40 g cm^**−**2^ Al
^12^C	0.128	0.240	0.423	0.667
^16^O	0.141	0.261	0.455	0.702
^24^Mg	0.160	0.295	0.503	0.753
^28^Si	0.169	0.309	0.522	0.772
^56^Fe	0.213	0.381	0.617	0.853

### The Role of Fragmentation in Space Radiation Protection

As the preceding has shown, fragmentation of primary GCR heavy ions as they traverse the hull of a spacecraft strongly influences the radiation environment inside. The flux of heavy ions is reduced behind shielding, which may result in a reduction in dose and certainly results in a reduction in dose equivalent. Because much of the uncertainty in the biological response to space radiation is due to uncertainty in response to heavy ions, the reduced flux of these ions behind shielding may also reduce some of the uncertainty in risk estimation.

It follows from the above considerations that an effective shield against GCRs is one that efficiently breaks up heavy incident ions into lighter ions. Put another way, we would expect materials with the largest cross sections per unit mass to be the best shields against GCRs. An important calculation verifying this was carried out by Wilson et al. (31), who found that a pure hydrogen shield would be extremely effective at reducing the dose equivalent from GCRs, and that the performance of other materials worsens with increasing atomic number. These calculations inspired subsequent experimental work (32, 33) that confirms the effectiveness of hydrogen in fragmenting heavy ions. It has subsequently been pointed out that the reductions of dose and dose equivalent at a point surrounded by hydrogenous shielding materials may be largely offset by the further transport of the components of the radiation field into a human body. That is, the fragmentation products of both proton–nucleus and nucleus–nucleus collisions, which include neutrons and low-energy protons, deposit doses of high-LET radiation inside the body that may be comparable to those from unattenuated GCR heavy ions, in terms of biological effect.

Despite these complications, point measurements and calculations of dose and dose equivalent are still important for characterizing the radiation environment to which crew members are exposed. In particular the effects of fragmentation can be assessed in terms of the average radiation quality of the field at a particular point behind shielding. In the methodology prescribed by ICRP 60, the average quality factor is given by < *Q* > = H/D, where *H* is the dose equivalent and *D* the dose. The dose and dose equivalent are given by

$$D=\frac{1}{\rho }\int \frac{d\Phi }{\text{dL}}L\text{dL and }H=\frac{1}{\rho }\int \frac{\text{d}\Phi }{\text{dL}}\text{LQ}(L)\text{dL}$$

where dΦ/dL is the differential fluence and the quality factor *Q* is solely a function of the LET, *L*, and ρ is the density of the target material in units of g cm^−3^. NASA has revised the ICRP 60 quality factors (34) with separate factors for solid tumors and leukemia. The NASA quality factors depend on the effective charge and velocity of the ion according to (Z^*2^/β^2^), rather than LET, a change intended to represent track structure. However, for ease of calculations, and making use of our existing analysis tools, we use the more familiar ICRP 60 *Q*(*L*) in the following. It is also notable that, subsequent to the publication of the revised NASA quality factors, analysis by Borak et al. (35) showed that the (Z^*2^/β^2^) dependences could be re-cast as LET dependences with only minor differences in the results for several space environment scenarios. The study was motivated by practical concerns about the difficulties of accurately measuring ion velocities at relativistic speeds using compact space-borne detectors.

As mentioned above, in free space, < *Q* > takes on values between 6 and 7, depending on the phase of the solar cycle. Considering that roughly 99% of GCRs are hydrogen or helium nuclei with *Q* = 1, this relatively large average value is remarkable. However, as will be shown in the next section, < *Q* > can be somewhat reduced by moderate depths of shielding.

### Measurements and Calculations for Space

A large body of experimental data has been obtained in LEO, using detectors flown on the Mir Station, Space Shuttle, and the International Space Station (ISS). Historically, many of the measurements have been made using passive detectors, which integrate over all contributions. In the case of LEO, this means passive detectors record a < *Q* > value that is the dose-averaged combination of the GCR and trapped radiation. This does not provide sufficient information to assess the effect of shielding on GCR < *Q* > values. For that, we must use data from active detectors, such as DOSTEL (36, 37), that have time resolution and which, therefore, allow for separate < *Q* > measurements for GCR and trapped particles. Recent DOSTEL measurements from ISS indicate a GCR < *Q* > in the vicinity of 3.1 in the Columbus module. This is quite comparable to results obtained by the Radiation Assessment Detector (RAD) detector (38), which measured a < *Q* > of 3.82 ± 0.25 during the transit for Earth to Mars in 2011–2012 (39) inside the modestly shielded Mars Science Laboratory spacecraft, and a value of 3.05 ± 0.30 on the surface of Mars (40) under somewhat more shielded conditions. Because the dose rate is far less affected by shielding than is dose equivalent (owing mainly to the production of secondary radiation in the shield), the reduction in < *Q* > is the main benefit of shielding.

Simple model calculations performed with PHITS give us some insight into the important characteristics of the GCR radiation field behind 20 g cm^−2^ of aluminum, which might be a typical shield for a human-crewed vehicle going into deep space. In this example, the GCR was treated as a pencil beam and shot at an aluminum target of the desired depth (7.4 cm). Particles crossing a cylindrical void downstream of the target were scored; the scoring region was 10 cm in diameter, large enough to contain the vast majority of particles emerging in the forward direction from the target. The void was separated from the downstream edge of the target by 1 mm of air, which stops extremely low-energy particles exiting the target. Although the simulated beam geometry, with a small parallel beam and a large detector, is not a realistic representation of the space environment, the scoring region used was large enough to capture the vast majority of particles exiting the target. This was tested with a simulated aluminum target 20 g cm^−2^ in depth; it was found that increasing the lateral dimensions of both the target and scoring regions by factors of two (a factor if 4 increase in areas) increased the number of scored charged particles by 3.3%. A total of 5 × 10^5^ simulated events were run, sufficient to make statistical errors negligible in the analysis. For computing dosimetric quantities, only particles with at least 10 keV/nuc of kinetic energy were scored. (This cut excluded <0.1% of neutrons and about 0.05% of charged particles.)

Initial charge and energy distributions were based on the Badhwar–O’Neill GCR flux model (41), for a solar modulation potential corresponding roughly to average conditions in 2014 and 2015, the most recent (weak) solar maximum. The GCR energy spectrum is harder at solar maximum than at solar minimum, i.e., relatively fewer low-energy ions are present due to the shielding effect of the interplanetary magnetic field. We begin the discussion by re-examining an aspect mentioned above, the re-population of ion species by “feed-down” from fragmentation of heavier ions. Table 2 shows, for the same ion species shown in Table 1, the expected losses due solely to fragmentation (based on energy-independent cross sections as per Table 1) and the predicted total attenuation of particle of that species, integrated over all incident energies using a more complete representation implemented in PHITS. The increased attenuation losses compared to those from fragmentation alone come about because some of the lower-energy GCR ions lose all their energy via ionization and come to rest in the shield. The picture of attenuation changes considerably when we consider only high-energy ions, as in the far-right column of Table 2. When incident ions with energies below 700 MeV/nuc are excluded (because many of them stop in 20 g cm^−2^ of aluminum), the number of carbon ions found after the target (counting ions of all energies) is actually greater than the number of incident by about 2%. This is due to feed-down from heavier species. There is a weaker, but not negligible, effect for the higher-*Z* GCRs, e.g., high-energy oxygen ions are only about one-third as depleted as would be expected simply based on fragmentation losses, etc. The results in Table 2 also include the (presumably small) effects of energy dependence in the nuclear cross sections.

**Table 2:** **Calculated attenuation and re-population of GCR ion species using the PHITS model to simulate transport through aluminum**.

Species	Charge-changing interaction probability in 20 g cm^**−**2^ Al	Attenuation in 20 g cm^**−**2^ Al (PHITS) including losses from ranging out	Attenuation in 20 g cm^**−**2^ Al (PHITS), *E*_incident_ **>**700 MeV/nuc
C	0.42	0.43	**−**0.02
O	0.46	0.49	0.17
Mg	0.50	0.59	0.30
Si	0.52	0.61	0.36
Fe	0.62	0.69	0.51

### Effects of Fragmentation on Dose and Dose Equivalent

Because a large proportion of GCRs have high energy, they are capable of producing large multiplicities of secondary particles as they traverse a spacecraft hull. These secondary particles are generally lower in LET than the primaries that created them. The net result tends to be (depending somewhat on the shield material and its depth) that dose is only slightly changed by the shield, but dose equivalent may be reduced significantly through the reduction in < *Q* > . Table 3 shows some results from the simulation described above, with a narrow beam of 10^6^ GCR-like ions incident on a 20 g cm^−2^ aluminum target.

**Table 3:** **Fluence and dose from PHITS simulation of GCRs on 20 g cm^−^^2^ Al**.

	Number of charged particles (*N*)	Average LET_**∞**_ in water (keV/**μ**m), **<***L***>**	*N* **×** **<***L***>**	**<** *Q* **>**
Incident beam	10^6^	0.700	7.0 × 10^5^	6.55
After Al target	1.16 × 10^6^	0.534	6.2 × 10^5^	3.77

The fractional change in dose from charged particles is simply the ratio of the *N* × <*L*> products, which works out to about a 12% decrease. There is an additional contribution to dose and dose equivalent from neutrons. In this example, we estimated the neutron contributions using conversion factors given in ICRP Publication 74 (42), in broad energy bins. The yield of neutrons is large, about 0.5 per incident GCR ion, making the statistical errors in the following quite small. Both dose and dose equivalent contributions of neutrons are on the order of 2% of the totals, so that the overall decrease in dose is roughly 10%. The dose equivalent from all particles behind the target is reduced from the incident dose equivalent by nearly 50%, driven mainly by the change in < *Q* >. Interestingly, the value of < *Q* > found in this simulation is very close to the value of 3.8 found during the Mars transit measurement made by MSL-RAD, which was under highly inhomogeneous shielding that averaged roughly 20 g cm^−2^. The shielding in that instance was a mix of materials, including tanks of hydrazine fuel that powered MSL’s descent vehicle.

The PHITS results were checked against the HZETRN code as implemented in the NASA OLTARIS tool (43). HZETRN predicts about a 40% reduction for this depth of aluminum compared to the 50% reduction predicted by PHITS. Dose results showed a qualitatively similar trend, as OLTARIS predicts a 10% increase in dose behind the target. Lastly, OLTARIS predicts a < *Q* > of 3.5 behind the shield, indicating that the bulk of the disagreement is likely to arise in the simulated multiplicities of low-LET particles, of which OLTARIS predicts a greater number. These drive up dose and to a lesser extent dose equivalent, while driving < *Q* > to a lower value. The larger dose but smaller < *Q* > result in nearly equal dose equivalent estimates from the two models.

These results are, in principle, dependent on the GCR flux model used. Even with a given model, results will vary as a function of the solar modulation specified for the calculation. An additional important caveat to the results above is that the simulations lacked a “back wall.” That is, the target is followed by the scoring volume, with nothing additional downstream. If a second wall is added (a geometry significantly more like a spacecraft or surface habitat), the effects of neutrons and other particles backscattered from the back wall appear to be significant (44).

## Nuclear Interactions of Carbon Beams

The effects of nuclear fragmentation that were elucidated in the discussion of space radiation shielding are, of course, also at work in heavy-ion radiotherapy. Fragmentation of heavy ions reduces < *Q* > at points behind modest depths of shielding in space, and this is generally desirable. It is, however, an undesirable effect in treatment, where one would like to have the highest possible biological effectiveness at the treatment site. Furthermore, as the above discussion highlighted, hydrogen is uniquely effective in terms of the fragmentation it causes per unit mass, and while this may eventually lead to the use and/or development of hydrogenous shields for space, it means that the high hydrogen content of healthy tissues in the entrance region efficiently fragments treatment beams.

### Carbon Beam Bragg Curves

**Figure 4:**
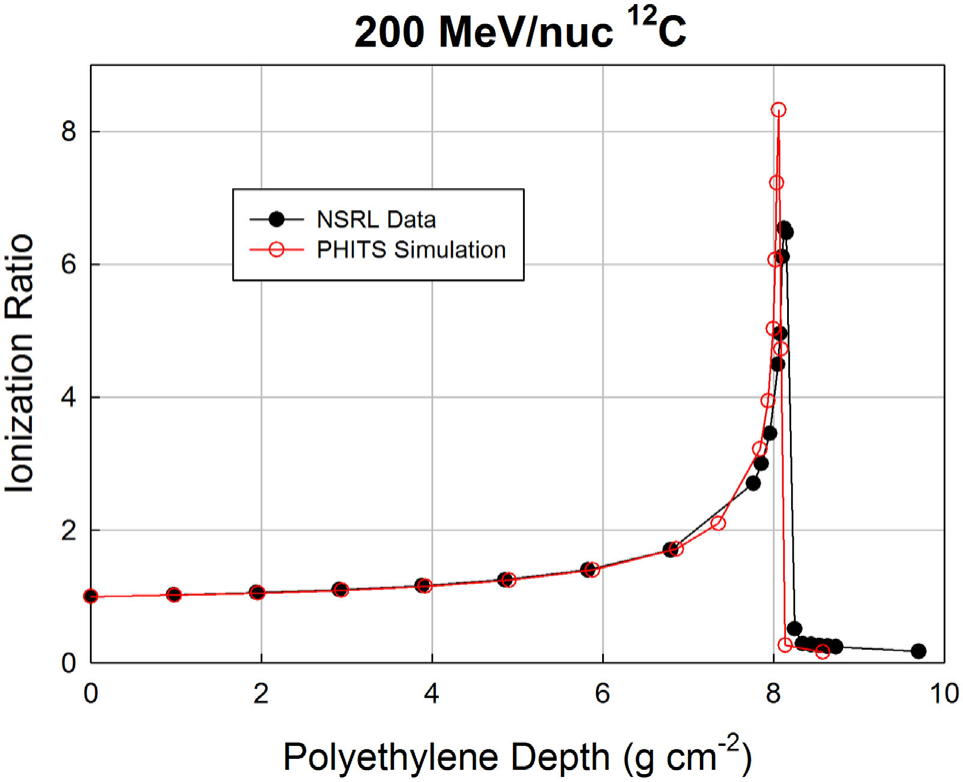
**Bragg curve data from NSRL and PHITS simulation for a 200 MeV/nuc ^12^C beam**.

The Bragg curves shown above for 200 and 293 MeV/nuc ^12^C were simulated, again using PHITS. In these simulations, the beamline geometry has a better correspondence to the treatment situation than does the simulation in which the GCR was treated as a pencil beam. It should be borne in mind that actual treatment planning makes use of spread-out Bragg peaks in order to treat finite tumor volumes. Furthermore, the simulations performed here did not represent the NSRL beamline in great detail. On the real beamline, the incident beam enters through a thin window, traverses an air gap, and enters the first ionization chamber. All ionization chambers have thin but finite entrance and exit windows, as well as foils that are not represented in the simulation, nor are the air gaps. Finally, the beam energy is not known precisely, and is actually inferred from the Bragg curve measurement. The fidelity of the simulation is, therefore, not perfect. Nonetheless, interesting trends are observed, as can be seen starting with Figure 4, which shows measured and simulated ionization ratios as a function of polyethylene target depth.

**Figure 5:**
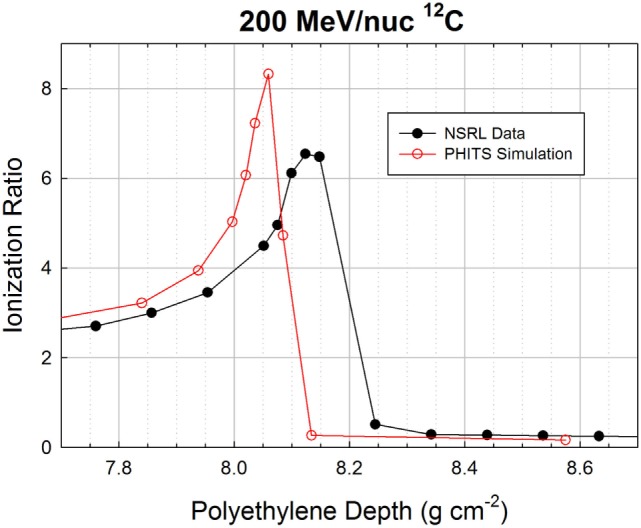
**Zoomed-in Bragg curve in the peak region of the 200 MeV/nuc ^12^C beam**.

Agreement over the first 7 cm is excellent, but slight deviations begin to appear beyond that point, as the surviving carbon ions and heaviest fragments slow down and approach the ends of their ranges. The peak ratio in the simulation occurs slightly before that in the data (8.06 g cm^−2^ in the simulation, 8.13 g cm^−2^ in the data), and has a higher value (8.3 vs. 6.5). The discrepancies are more visible in Figure 5, which zooms in on the peak region. The distal edge appears to be less populated in the simulation than in the data, which is consistent with the simulation slightly underestimating the probability of fragmentation as the carbon ions traverse the target. If the model had a higher nuclear cross section, it would both reduce the peak value of the ionization ratio and increase the population of fragments in the distal edge.

The location of the simulated peak is slightly offset from the peak location in the data, by <1%. This could easily be an artifact of the inaccuracies of the simulated beamline, and/or a slight difference in beam energy between the nominal 200 MeV/nuc and the actual energy. If the difference between the measurement and simulation is attributable only (or dominantly) to the initial energy, the required extra range would be fully accounted for if the beam energy was 200.5 MeV/nuc, and in fact the NSRL team estimates the beam energy to have been 200.2 MeV/nuc (though 200.0 MeV/nuc was used in the simulation).

**Figure 6:**
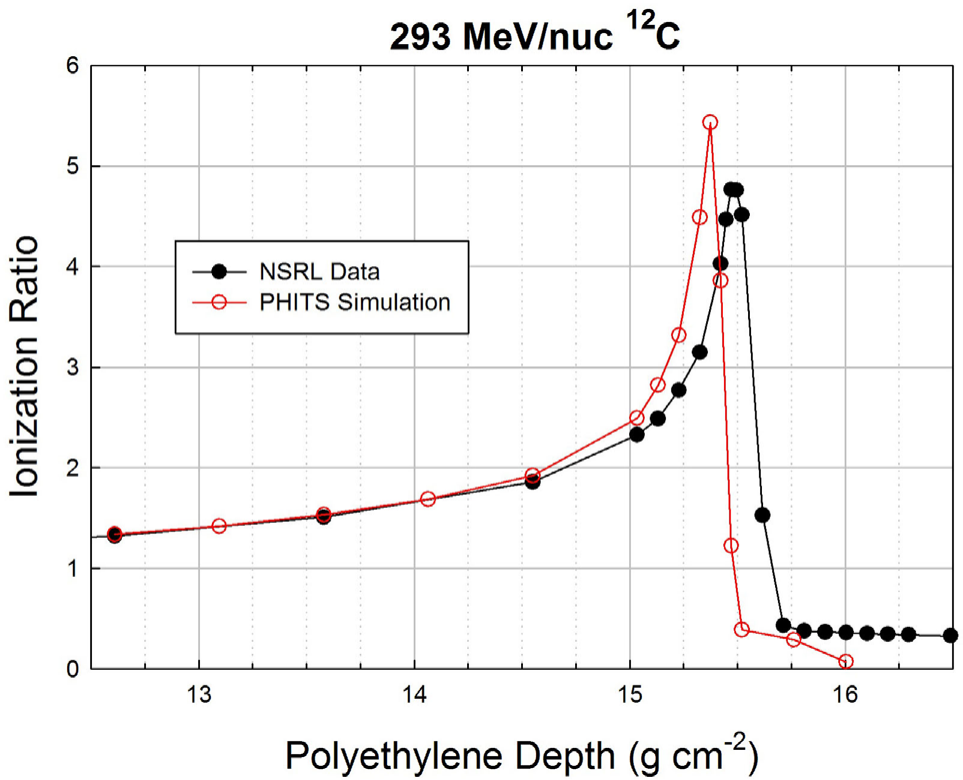
**Zoomed-in Bragg curve in the peak region of the 293 MeV/nuc ^12^C beam**.

Finally, in Figure 6, we show data and simulations in a 4-cm region of the Bragg peak region for the 293 MeV/nuc ^12^C beam. The differences between the data and simulation are qualitatively the same at this energy as at the lower energy: the peak is again slightly shifted to a smaller depth in the simulation (15.37 vs. 15.95 g cm^−2^) and again has a higher peak value in the simulation (5.43 vs. 4.77). The higher peak values in the simulation vs. data for both ^12^C energies are likely due to having simulated a perfectly monoenergetic incident beam, whereas the real beam has a finite momentum spread due to the optics of the beamline transport system.

The distal edge, which is populated mostly by hydrogen and helium ions (including a significant share of ^2^H), falls off much more rapidly in the simulation than in the data. It is possible that the differences in the distal edge could be due to the limited radius of the cylindrical volume used in the simulation to score particles exiting the target, which was set to 10 cm, far wider than the pencil beam diameter (1 cm). Some of the simulated exiting particles were likely more than 10 cm from the beam axis and, hence, were not scored, whereas the actual ionization chambers that were used to obtain the data are much wider.

Our broader purpose here is not to diagnose possible shortcomings in the model or the beamline, but rather to show that even with a fairly crude simulation of the beamline, fragmentation and energy loss effects can be modeled with good fidelity for a therapy beam. The simulations also give insight into the composition of particles in the rising edge, Bragg peak, and distal edge of the beam. The simulations indicate that there are roughly equal numbers of H and He ions in the distal edge, of which the He ions contribute approximately 80% of the dose. In the peak region, about 45% of the charged ions are carbon ions that survive traversal through nearly 16 g cm^−2^, well in line with the 50% estimate given above, especially since here the total count of particles includes fragments that are generally produced with multiplicities >1. The remaining particles consist of about one-third helium ions, 10% hydrogen ions, 7% boron, with the remainder divided more or less equally between lithium and beryllium. When higher-energy beams are used in treatment, the fraction of carbon ions in the Bragg peak region is even lower than this.

### Geometric Cross Sections

**Figure 7:**
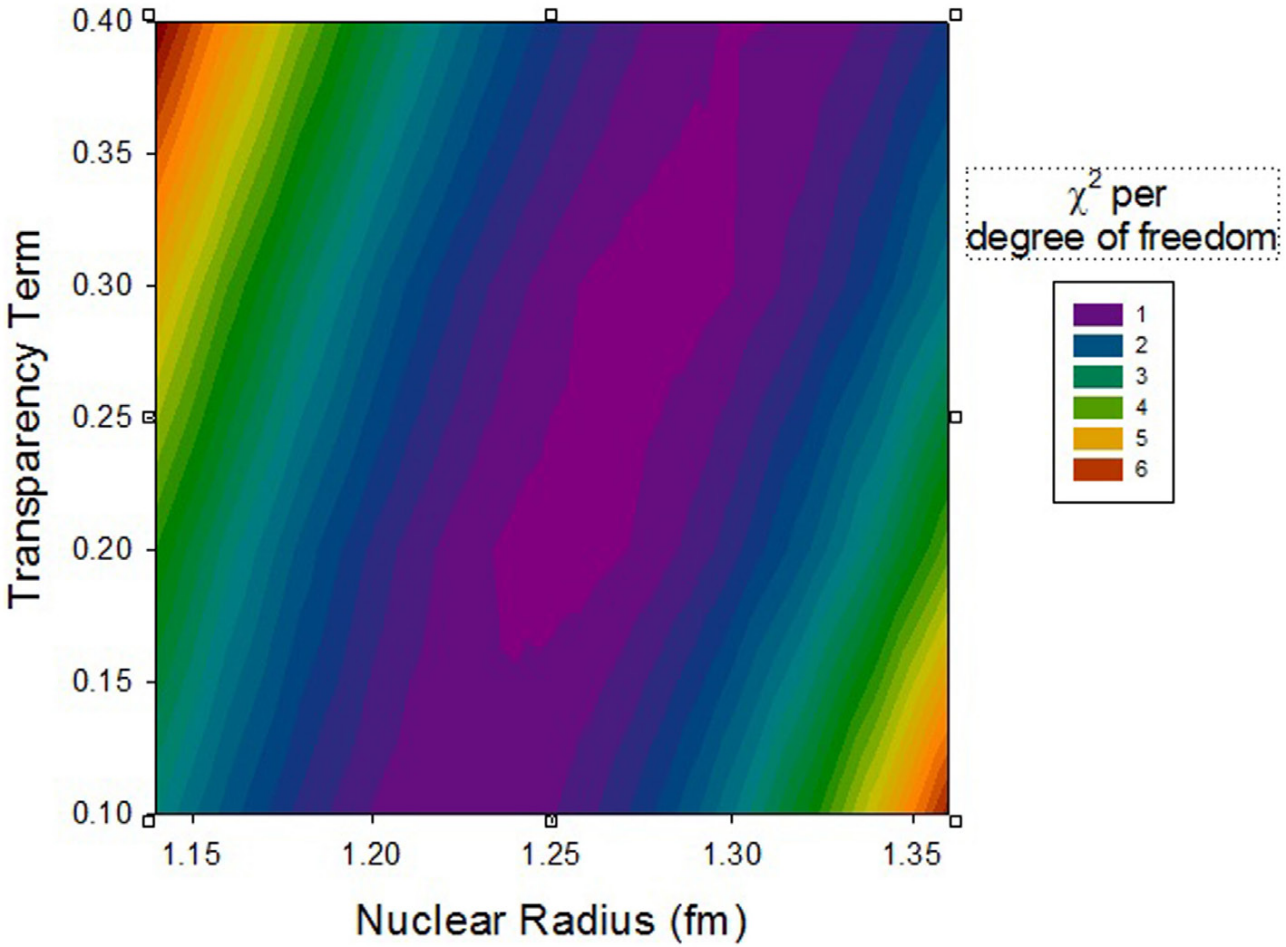
**Contour plot of χ^2^ for varying nuclear radius and transparency parameters in the geometric cross section formula**.

A fairly large collection of nuclear cross section data was obtained in our previous experiments. Most but not all results have been published (16, 19–21). Projectiles included ^4^He, ^10^B, ^12^C, ^14^N, ^16^O, ^20^Ne, ^24^Mg, ^28^Si, ^40^Ar, ^48^Ti, and ^56^Fe. Target data for H, C, Al, Cu, Sn, and Pb were obtained. Beam energies ranged from 230 to 1200 MeV/nuc, a range in which the approximation of energy-independent cross sections appears to be valid for targets other than H. Data were obtained for both total charge-changing cross sections and fragment production cross sections with no isotopic resolution. Here, we look at the measured charge-changing cross sections in comparison with the Wilson–Townsend formula given above, treating the nuclear radius (nominally 1.26 fm) and the transparency term (nominally 0.2) as adjustable parameters. A series of χ^2^ values was calculated for agreement between the data and model, and is shown as a contour plot in Figure 7 where the color indicates the level of agreement. Though the uncertainties on the charge-changing cross sections are typically on the order of ±3 to 5%, we have inflated them here to ±10% to obtain reasonable χ^2^ values on the order of 1 per degree of freedom for the best fits. Clearly, the strong correlation between parameters yields relatively poor constraints. Equally good combinations occur in the range from 1.25 to 1.30 fm with transparency terms varying from 0.2 to 0.4 depending on the nuclear radius value.

For all parameter variations tried here, the majority of the χ^2^ comes from data taken with the lighter targets (H, C, Al), while the agreement is substantially better with the heavier targets (Cu, Sn, Pb). If the search for minimum χ^2^ is limited to just the light targets, the best-fit parameters are 1.235 fm for the nuclear radius and 0.16 for the transparency term.

A qualitatively similar analysis effort was undertaken by Heckman et al. (45) using nuclear emulsion data, with fits to the most basic form of the Bradt–Peters geometric cross section model. Across a range of projectile/target masses, a consistent value of the nuclear radius, *r_*0*_*, was found (1.36 ± 0.02 fm), but a highly variable value of *b* was needed to fit the data. Of particular interest, for ^4^He projectiles, a very large *b* of 1.10 ± 0.04 was found, indicating fragmentation cross sections smaller than expected from the geometry of heavier ions. A likely explanation is the tight binding of the nucleons in ^4^He, so that these ions are less likely than others to break up when undergoing peripheral collisions. In view of the deleterious effects of fragmentation in the treatment setting, this seems to suggest that ^4^He might be a particularly good ion to use in therapy. The measured charge-changing cross sections on H and C show that the mfp of ^4^He in polyethylene is 66 g cm^−2^, so that at a penetration depth of 16 g cm^−2^ (the Bragg peak location of the 290 MeV/nuc ^12^C beam), some 78% of the ^4^He is still intact, compared to about 50% for ^12^C. The corresponding energy for ^4^He to stop at the same depth is about 155 MeV/nuc. Because of the difference in charge (2 vs. 6), the peak ionization ratio would be expected to be lower than the value of about 5 found for ^12^C; a PHITS simulation suggests that the peak value would be between 4 and 5. This may be a worthwhile tradeoff given that the lateral and distal doses would be significantly less than they are with carbon beams.

## Conclusion

Nuclear fragmentation is an important phenomenon both in space radiation protection, where it reduces exposure to heavy ions with high biological effectiveness, and in heavy-ion therapy where it dilutes the effectiveness of the primary beam ion and causes dose to be deposited outside the treatment volume. Previous code comparisons, along with the simulations and comparisons to beam and flight data shown here, give us confidence that current Monte Carlo codes are able to predict the combined effects of fragmentation and energy loss both in space and in carbon ion therapy with good fidelity. The small fragmentation cross section of ^4^He suggests that it may be a particularly useful ion for therapy.

## Author Contributions

CZ did the majority of writing and the transport model calculations used in the paper. CT provided the NSRL experimental data and did a significant share of the writing, and proofread and corrected the parts written by CZ. The basic idea for the article grew out of discussions between the two authors about the pitfalls of carbon ion therapy and the quest to find the optimal treatment ion species for radiation therapy.

## Conflict of Interest Statement

The authors declare that the research was conducted in the absence of any commercial or financial relationships that could be construed as a potential conflict of interest.
